# Multimodal evaluation of the cerebrovascular reserve in Neurofibromatosis type 1 patients with Moyamoya syndrome

**DOI:** 10.1007/s10072-020-04574-4

**Published:** 2020-07-10

**Authors:** Alessandra D’Amico, Lorenzo Ugga, Sirio Cocozza, Sara Maria delle Acque Giorgio, Domenico Cicala, Claudia Santoro, Daniela Melis, Giuseppe Cinalli, Arturo Brunetti, Sabina Pappatà

**Affiliations:** 1 Department of Advanced Biomedical Sciences, University of Naples “Federico II”, Via Pansini, 5, 80131 Naples, Italy; 2 Department of Pediatric Neurosurgery, Santobono-Pausilipon Children’s Hospital, Naples, Italy; 3 Referral Centre of Neurofibromatosis, Department of Woman and Child, Specialistic and General Surgery, University “Luigi Vanvitelli”, Naples, Italy; 4 Department of Translational Medical Sciences, Section of Pediatrics, University of Naples “Federico II”, Naples, Italy; 5 Institute of Biostructure and Bioimaging, National Research Council, Naples, Italy

**Keywords:** Moyamoya, MRI, SPECT, Neurofibromatosis type 1

## Abstract

**Purpose:**

Moyamoya syndrome (MMS) is a rare intracranial arterial vasculopathy which can occur in neurofibromatosis type 1 (NF1) disease, representing a cause of cerebrovascular reserve (CVR) impairment, possibly leading to ischemic stroke. Here, we evaluated noninvasive imaging techniques used to assess CVR in MMS patients, describing clinical and imaging findings in patients affected by MMS-NF1.

**Methods:**

Following strict inclusion and exclusion criteria, in this retrospective observational study, we evaluated imaging data of nine consecutive MMS-NF1 patients (M/F = 5/4, mean age: 12.6 ± 4.0). Subjects underwent a multimodal evaluation of cerebral vascular status, including intracranial arterial MR Angiography (MRA), MRI perfusion with dynamic susceptibility contrast (DSC) technique, and 99mTc-hexamethylpropyleneamine oxime (HMPAO) SPECT.

**Results:**

In 8 out 9 patients (88.8%, 6/8 symptomatic), time-to-peak maps were correlated with the involved cerebral hemisphere, while in 6 out 9 patients (66.6%, 5/6 symptomatic), mean transit time (MTT) maps showed correspondence with the affected cerebrovascular territories. Cerebral blood flow (CBF) calculated using DSC perfusion failed to detect the hypoperfused regions instead identified by SPECT-CBF in all patients, while MTT maps overlapped with SPECT-CBF data in all cases and time-to-peak maps in 60.0%.

**Conclusions:**

Although SPECT imaging still represents the gold standard for CBF assessment, our results suggest that data obtained using DSC perfusion technique, and in particular MTT maps, might be a very useful and noninvasive tool for evaluating hemodynamic status in MMS-NF1 patients.

**Electronic supplementary material:**

The online version of this article (10.1007/s10072-020-04574-4) contains supplementary material, which is available to authorized users.

## Introduction

Neurofibromatosis type 1 (NF1) is a multisystem autosomal dominant disorder caused by mutations in the neurofibromin tumor suppressor gene, mainly affecting eyes, skin, bones, and central nervous system (CNS) [1, 2]. Cerebral arterial involvement is a well-recognized feature of this condition, mostly related to vessel stenosis (2.5–6% of cases) [3, 4], although less frequent manifestations such as aneurysms or artero-venous malformations and fistulas could be present in this disease [2]. Indeed, the most frequent expression of vascular involvement in NF1 patients is a progressive and significant arteriopathy similar to those observed in moyamoya (MM) disease (MMD), regarded as MM syndrome (MMS) [5]. These conditions share a similar diagnostic workflow, clinical presentation, and outcome after surgical revascularization [6], with MMS being defined when MM occurs in association with a well-recognized condition (such as NF1), while subjects without known associated risk factors are classified as MMD patients [5]. Nevertheless, both conditions are characterized by a progressive intimal proliferation resulting in luminal obstruction, mostly affecting the supraclinoid internal carotid arteries (ICAs) and the proximal segment of both anterior and middle cerebral arteries (ACA, MCA). Similarly to what described for MMD, in MMS, the development of tortuous leptomeningeal collateral networks and compensatory dilations of perforating arteries produces the typical angiographic image of “puff of smoke” [7–9]. Despite digital subtraction angiography (DSA) is still considered the gold standard for diagnosis and presurgical evaluation of both MMD and MMS, MR angiography (MRA) represents a valuable diagnostic tool in these conditions [10]. Indeed, according to the “Research Committee for the Diagnosis of MMD in Japan” guidelines [10], cerebral DSA is not mandatory if MRA demonstrates ICA or proximal ACA/MCA stenosis. Moreover, abnormal vascular network with flow voids in the basal ganglia and, especially, in the Sylvian fissures on T2-weighted images strengthens the diagnosis of MMD and MMS [11].

Although different grades of hemodynamic insufficiency occur in these conditions, clinical symptoms might or might not be present when diagnosis is reached [12]. Usually, the initial manifestations are related to the occurrence of ischemic events, often multiple and recurrent due to the development of the steno-occlusive lesions hallmark of this condition [13]. Transient ischemic attacks have also been reported, especially in pediatric population [14], while hemorrhagic events are more common in adults [15]. Along with symptoms related to the occurrence of ischemic events, patients can also present with headache, which has been reported to be a common clinical finding of this condition [16] that may improve after a successful revascularization surgery [17]. For these reasons and to avoid serious and invalidating complications, it is important to immediately recognize MMS. In this light, both single-photon emission computed tomography (SPECT) with acetazolamide challenge and positron emission tomography (PET) examinations represent valuable tools to assess cerebrovascular reserve (CVR) and hemodynamic impairment in MM patients, providing quantitative measures of different cerebral perfusion variables [18, 19]. These include the relative cerebral blood flow and volume (rCBF and rCBV, respectively), the oxygen extraction fraction (OEF) and the regional cerebral metabolic rate for oxygen (rCMRO_2_), all parameters that help in selecting patients at higher risk of stroke and therefore requiring a revascularization surgery, given their association with severe hypoperfusion and marked hemodynamic failure [20].

Nevertheless, these imaging procedures are known to be relatively invasive, exposing young patients to ionizing radiation. For this reason, in recent years, less invasive perfusion techniques using MRI have been proposed, such as arterial spin labeling (ASL), dynamic susceptibility contrast perfusion weighted imaging (DSC-PWI), or CO_2_-triggered blood-oxygen-level-dependent (BOLD) functional MRI, reported to have a similar effectiveness to evaluate CVR in both MMD and MMS patients [21, 22]. Indeed, it has been recently reported that MR-derived perfusion parameters (namely, the mean transit time (MTT)) negatively correlated with CVR measured with SPECT and acetazolamide challenge in MMD patients, suggesting that DSC-MRI may provide valuable information about the CVR in these patients [21].

Given this background, the aim of the study was to expand the current knowledge about the evaluation of CVR in MMS-NF1 patients, by (i) describing clinical and imaging findings in subjects undergoing a multimodal imaging evaluation; (ii) investigating a possible role of noninvasive imaging techniques, such as DSC-PWI, in the evaluation of CVR in MMS-NF1 patients; and (iii) comparing SPECT and MRI derived cerebral perfusion parameters in a subgroup of patients.

## Material and methods

### Participants

In this retrospective observational study, we reviewed data of NF1 patients clinically evaluated between January 2007 and December 2017 at two Referral Centers (University “Luigi Vanvitelli,” Naples, Italy, and University “Federico II”, Naples, Italy). Inclusion criteria were the following: diagnosis of NF1 according to the recommendations of the National Institutes of Health [23], availability of MRI acquisition, diagnosis of MMS suspected on the MRA data according to the available guidelines [10], and availability of both MRA and DSC-PWI sequences. On the other hand, subjects with the presence of other neurological conditions extending beyond the spectrum of NF1 or with significant artifacts on the neuroradiological images were excluded from this work.

The study was carried out in compliance with the Helsinki Declaration, with all patients that provided a written consent to execution of the imaging exams and for any clinical research purposes. In case of subjects with less than 18 years, the legal guardians provided the required written consent.

### MRI data acquisition and processing

Brain MR scans were all performed on the same 1.5 T scanner (Gyroscan Intera, Philips Medical System, Best, Netherlands) at a single center. Along with clinical T1-weighted, T2-weighted, fluid attenuated inversion recovery (FLAIR), and diffusion weighted imaging (DWI) sequences, MR protocol included a 3D time-of-flight (TOF) MRA for the study of the circle of Willis (TR: 22 ms; TE: 7 ms; Flip Angle: 20°; matrix: 304 × 194; slice thickness: 1.4 mm) and a DSC-PWI sequence (TR: 760 ms; TE: 30 ms; Flip Angle: 40°; matrix: 128 × 128; slice thickness: 7 mm; 18 axial slices; 70 volumes; acquisition time: 100 s; temporal resolution: 1.5 s). Before the DSC-PWI sequence, a pre-bolus of 1 cc of Gd-DTPA (Gadobutrol, Gadovist®, Bayer) was administrated to correct T1-weighted leakage phenomenon. The DSC-PWI sequence was obtained as follows: 9 dynamic series were acquired before injection, followed by 61 volumes after administration of 0.1 mmol/Kg of Gd-DTPA and a saline flush of 25 mL (2.5 ml/s). DSC-PWI data were then processed offline using Olea Sphere MR perfusion software program, v.3.0 (Olea Medical, La Ciotat, France) with a Bayesian probabilistic method, with automated multiple arterial input function (AIF) selection, to obtain CBF, CBV, MTT, and TTP maps.

### SPECT-CBF data acquisition and processing

Data of rCBF from SPECT acquisitions were obtained after slow bolus intravenous injection of 51.8 MBq/Kg of ^99m^Tc-HMPAO (Ceretec®, Amersham, UK), according to the procedural guidelines of the European Association of Nuclear Medicine [24]. The radiotracer was injected with the patient lying in supine position with eyes closed in a dimly lit, quiet room. Cerebral activity was recorded in step-and-shoot mode using a dual-head gamma camera equipped with a general purpose, low energy, parallel-hole collimator (E-cam, Siemens Medical Systems). Images were acquired with a 128 × 128 matrix for 360 degrees evaluation with a circular orbit. A total of 60 frames were taken at 6-degree intervals of 30 s for each with a total acquisition time of 30 min. The data were reconstructed with filtered back projection using a Butterworth filter (cutoff 1, order 10), and corrected for attenuation using Chang’s algorithm on transaxial images (attenuation factor 0.120 cm^−1^). Coronal and sagittal slices were calculated with the original transaxial images.

### Image evaluation

To evaluate the ability of imaging techniques in the detection of vascular alterations in MMS-NF1 patients, images were analyzed as follows.

All MRI data were evaluated in consensus by two neuroradiologists with more than 8 and 20 years of experience in the field of neuroimaging, blinded to the clinical and nuclear medicine findings, and were asked to establish and report which cerebral artery was more affected by stenosis, also reporting abnormal signal regions on perfusion maps.

Similarly, SPECT images were reviewed in consensus by two experienced nuclear medicine physicians, also with more than 5 and 25 years of experience in the field of neuroimaging, blinded to the clinical diagnosis and MRI findings. Using the cerebellum activity as the reference region for visual inspection, hypoperfused cortical structures were identified.

Finally, to evaluate the correlation between MRI and SPECT data, the two most experienced neuroradiologist and nuclear medicine physicians evaluated in consensus both imaging data.

## Results

**Fig. 1:**
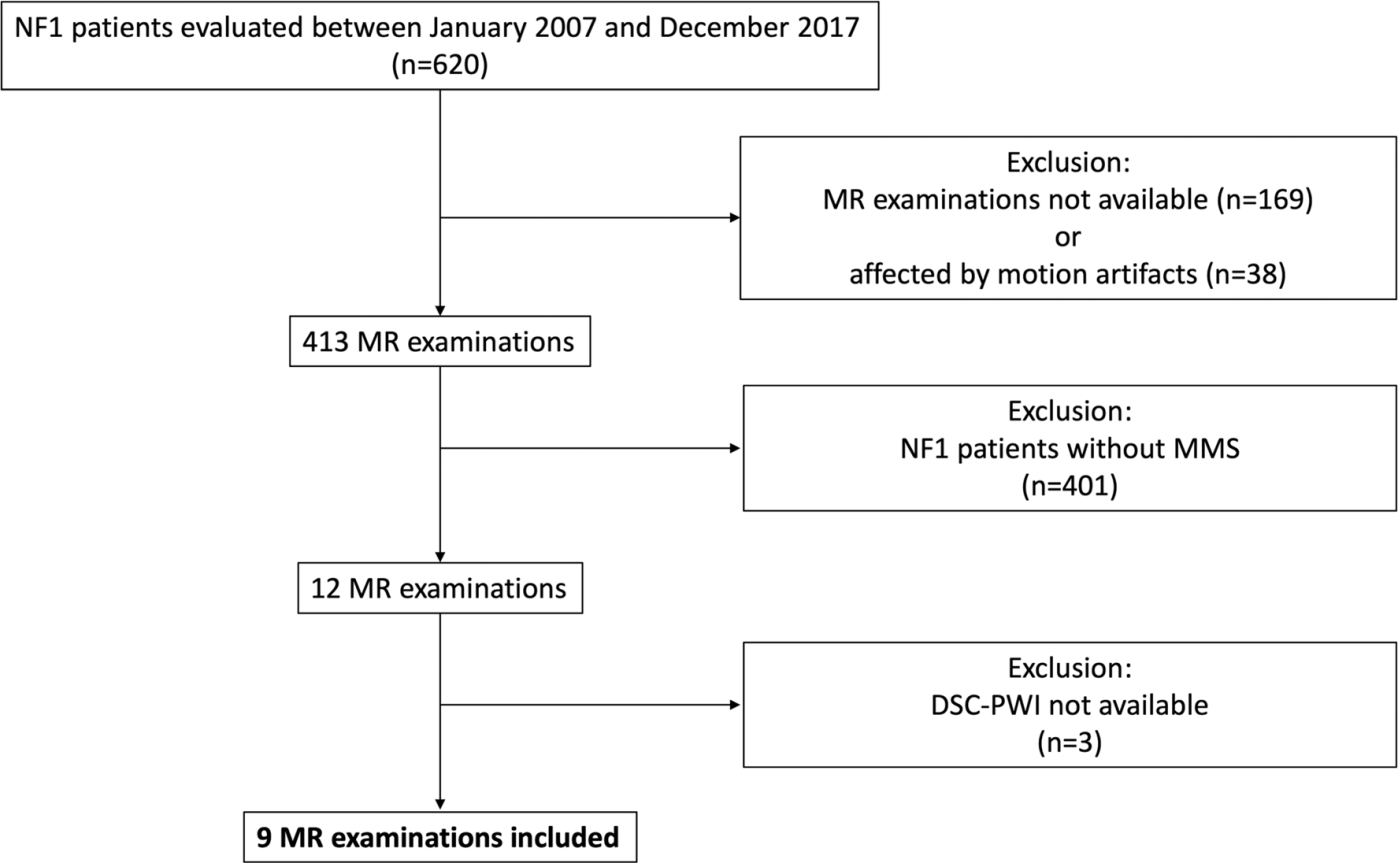
The flow diagram showing patient selection

Following inclusion and exclusion criteria (Fig. 1), from data available in a cohort of 620 NF1 patients, images of 9 subjects were evaluated in this study (M/F = 5/4, mean age: 12.6 ± 4.0 years, age range: 7–21 years) (Table 1). Furthermore, a SPECT-CBF scan, obtained within 3 months from the MRI, was available in 5 out of 9 patients. Finally, 4 subjects underwent a DSA before a surgical indirect revascularization by encephalo-duro-arterio-myo-synangiosis (EDAMS) procedure.

**Table 1:** Summary of demographic, clinical, and imaging data of the studied population

Patient	Age	Sex	Clinical data	MRA	SPECT-CBF	MRI-CBF	CBV	MTT	TTP	Treatment	Ivy sign/MM vessels
#1	11	M	Migraine; dizziness	Bilateral ICAs; bilateral ACAs; L PCA	↓ L hemisphere	Symmetric regular	↑L hemisphere	↑L parietal	↑L hemisphere	EDAMS	L hemisphere and R temporo-occipital ivy sign
#2	11	F	Asymptomatic	R ICA, R MCA	↓ R temporal, occipital	Symmetric regular	↑R hemisphere > parietal	↑R parietal, frontal, occipital and caudate	↑R parietal frontal occipital caudate	EDAMS	R temporo-parietal ivy sign; R MM vessels
#3	11	M	Headache	L MCA	Symmetric Regular	Symmetric regular	↑ L frontal	symmetric normal	↑L frontal insular parietal	EDAMS	L MM vessels
#4	14	M	Migraine	R ICA	↓ R temporo-occipital frontal	Symmetric regular	Symmetric regular	↑R temporo-occipital frontal	↑R temporal occipital frontal	Follow-up	–
#5	16	M	Mental delay	R MCA	Symmetric regular	Symmetric regular	Symmetric regular	Symmetric regular	↑R hemisphere	Follow-up	R MM vessels
#6	12	F	Headache, L paresthesia	Bilateral MCAs	–	Symmetric bilateral stenosis	Symmetric bilateral stenosis	Symmetricbilateral stenosis	symmetric (bilateral stenosis)	EDAMS	L MM vessels
#7	7	F	Asymptomatic	R PCA	–	↓ R occipital, R cerebellum	↓ R occipital, R cerebellum	↑ R occipital, R cerebellum	↑ R occipital, R cerebellum	Follow-up	–
#8	11	M	Asymptomatic	L PCA	–	symmetric normal	↑ L occipital	↑ L occipital	↑ L occipital	Follow-up	–
#9	21	F	Severe mental delay; epilepsy; L mild hemiparesis	R ICA	–	↓R anterior frontal (but ↑in the remaining hemisphere)	↓ R anterior frontal ↑R insular, posterior frontal, parietal	↑ R hemisphere	↑ R hemisphere	Follow-up	R hemisphere ivy sign; R MM vessels

At the MRI examination, all patients (100.0%) showed the presence of a vascular narrowing affecting either the ICA or the MCA, while in 3 subjects (33.3%), an involvement of the posterior circulation was also present. The presence of collaterals was found in 6 patients (66.7%), with an increase in CBV values at the level of the subarachnoid spaces of the ipsilateral stenotic artery that was found in 5/9 cases (55.6%), due to vessel compensatory dilation phenomena. On the other hand, in only 2/9 cases (22.2%), CBV maps showed a significant signal reduction due to chronic hypoperfusion, mainly associated to atrophy and gliosis, while in 3/9 patients (33.3%), no significant asymmetry was found on the CBV maps. Interestingly, in one subject, a complex pattern of cerebral perfusion was found, made of both increased and decreased signal within the same hemisphere. While MRI-CBF was not useful to detect hypoperfused regions, SPECT-CBF was decreased on the same side of the affected vessel in 3/5 patients (60.0%). Finally, MTT maps overlapped with the side of the affected cerebrovascular territory in 6/9 subjects (66.7%), while TTP maps were even more correlated with the affected side, showing a concordance in 8/9 of the cases (88.9%).

When SPECT imaging was performed, concordance of both CBV and TTP measured with MRI was found in 3/5 cases (60.0%). Interestingly, among all the DSC-PWI variables, the one providing the highest concordance with SPECT findings was the MTT, showing concordance in all cases (5/5 patients, 100.0%).

**Fig. 2:**
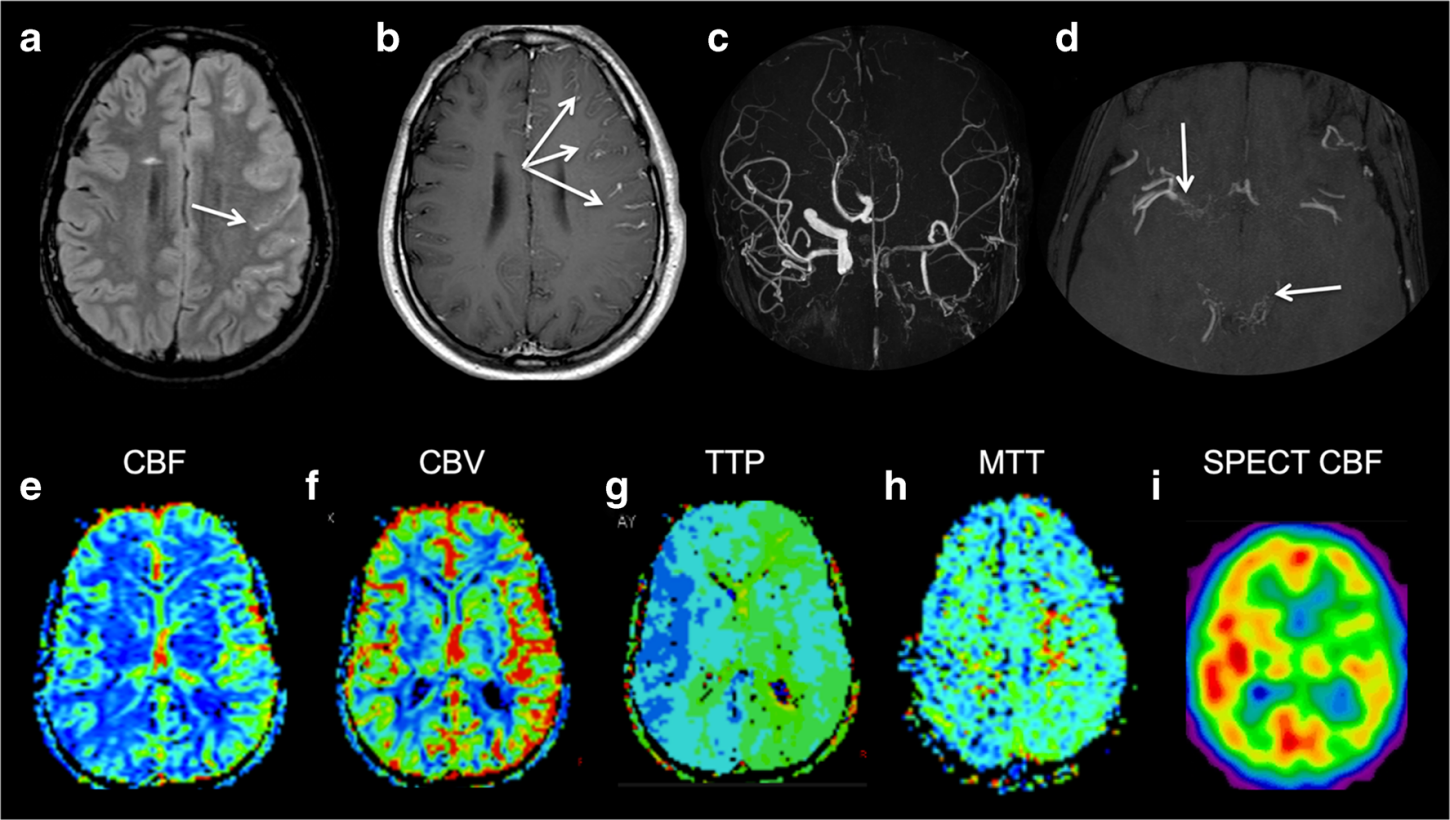
Imaging findings in patient #1. FLAIR image (**a**) shows “ivy sign” in the subarachnoid spaces of the left cerebral hemisphere (arrows) which corresponds to linear enhancement on contrast enhanced T1-weighted sequence (**b**). MR angiography maximum intensity projection (**c**) showing left ICA occlusion (related to the presence of a trigeminal neurofibroma infiltrating the cavernous sinus) and multiple MM stenoses on the right distal ICA, A1 segments, and left P3 and P4 segments. Multiple thin collaterals were observed in the right sylvian fissure and in the quadrigeminal cistern (arrows on **d**). On DSC-PWI, CBF (**e**) is symmetric, while CBV (**f**) and TTP (**g**) maps show an increase in the left hemisphere. TTP (**g**), with an increase in MTT (H) at the level of the left parietal lobe. Finally, SPECT demonstrates left cerebral hemisphere hypoperfusion (I)

**Fig. 3:**
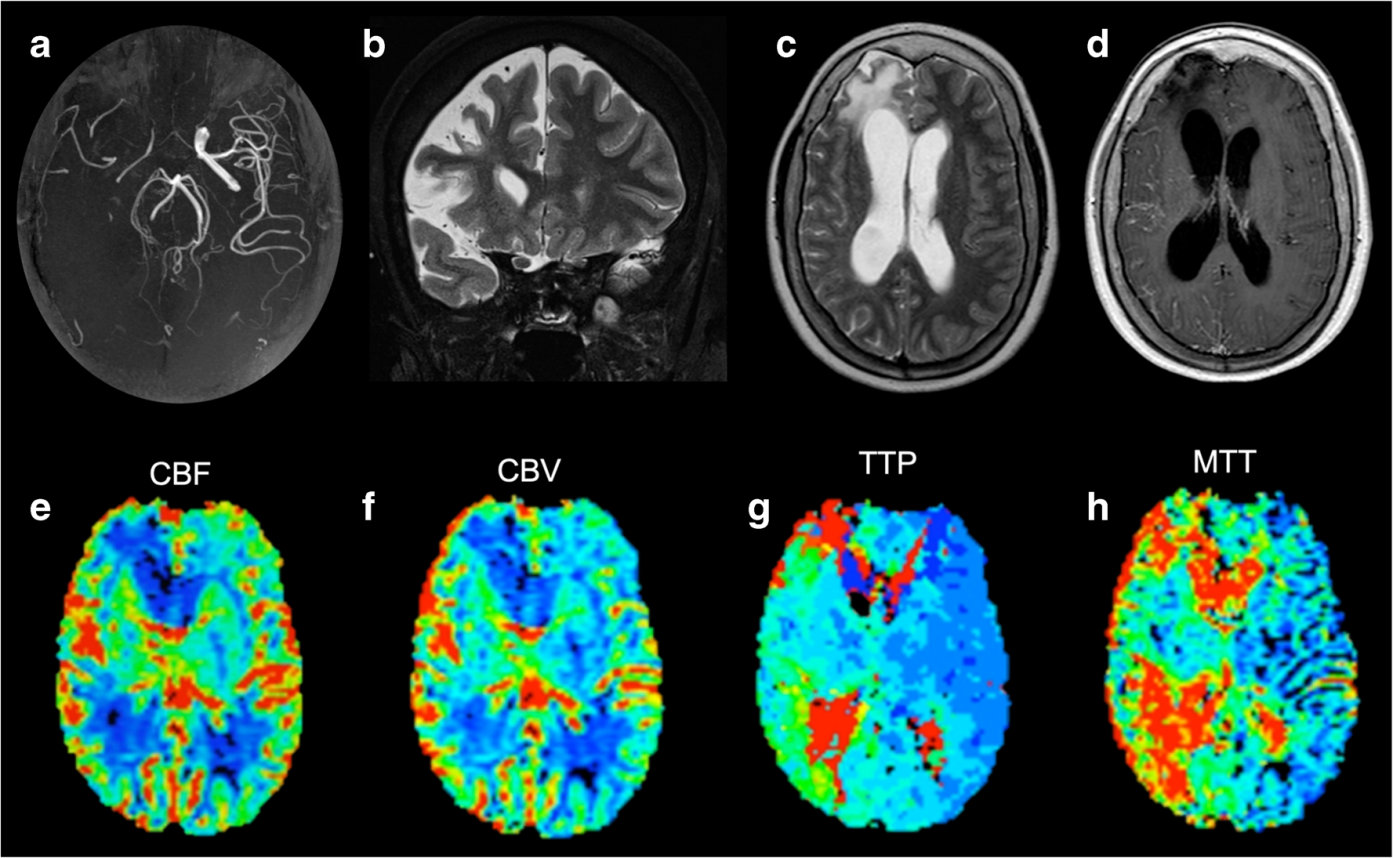
Imaging findings in patient #9. Occlusion of the right intracranial ICA is evident on MRA (**a**). The right frontal lobe appears shrunken and hyperintense on T2-weighted images (**b**–**c**), due to the chronic hypoperfusion. Contrast-enhanced T1-weighted sequence shows ipsilateral leptomeningeal enhancement and MM vessels (**d**). Reduction of CBF (**e**) and CBV (**f**) of the right atrophic frontal lobe was observed, with increase of the whole right hemisphere on TTP (**g**) and MTT (**h**) maps. Please note the marked signal increase on the same maps among the remaining right hemisphere

Two examples of imaging findings are shown in Figs. 2 and 3, while a complete description of all clinical and imaging subjects, with corresponding selected radiological features, is available in Supplementary Materials.

## Discussion

In this case series, we report 9 NF1 patients with vascular involvement undergoing MRI, MRA, and DSC-PWI, describing findings and correlations between these different parameters and reporting two cases of NF1 patients with primitive involvement of the vertebro-basilar system, a finding not described yet in literature, to the best of our knowledge. Furthermore, in 5 subjects, SPECT-CBF data were obtained, and we correlated cerebral perfusion parameters from different imaging techniques, suggesting a possible role of DSC-PWI and, in particular of MTT maps, in the radiological evaluation of these patients.

In NF1 patients, MMS is usually characterized by unilateral stenosis of distal ICA and its branches, whereas progression to bilateral involvement is reported in literature with a variable incidence (10–100%) [25]. Although both the anterior and the posterior circulations can be affected, the anterior system is usually more frequently and precociously involved [26, 27]. MRI, with particular reference to MRA, is a useful and noninvasive imaging technique that allows for the diagnosis and the follow-up of MMS-NF1 patients [25]. In particular, several authors have examined the accuracy of MRA in the evaluation of vascular involvement in NF1 patients, reporting a sensitivity and specificity for ICA, ACA, and MCA stenosis close to 100% [28].

In MM patients, dilation of compensatory pial and medullary vessel anastomosis plays a critical role to compensate the decreased brain perfusion pressure [29]. This phenomenon is recognizable on MRI, corresponding to subarachnoid hyperintensity on FLAIR images and leptomeningeal enhancement after gadolinium administration. These findings, known as “ivy sign” and “medullary streaks,” respectively [30, 31], positively correlate to the CVR reduction and to the onset of ischemic symptoms [32]. In our sample, of the 3 patients with both “ivy sign” and “medullary streaks,” 2 subjects showed clinical symptoms referable to cerebral hypoperfusion (patient #1, with migraine and dizziness, and patient #9, with history of epilepsy and left hemiparesis), while patient #2, despite being clinically asymptomatic, already developed an ischemic stroke in the right caudate nucleus, ipsilateral to the ivy sign and to the arterial stenosis.

Cerebral perfusion imaging is crucial in MM patients for the evaluation of hemodynamic variations, before and after surgery [33, 34]. Different MRI techniques can be used for the study of cerebral perfusion, including ASL and DSC-PWI [35]. Despite ASL represents a less invasive technique compared to DSC-PWI, given the absence of contrast administration, it is known to be limited by different factors. In particular, given that the time between labeling in the feeding arteries and the arrival of labeled blood in tissue (arterial transit time, ATT) have a significant effect on the ASL signal and in MMD, the ATT may be prolonged, this could lead to a focal intravascular signal artifacts, with subsequent underestimation of CBF [36]. This limitation, coupled to the notion that NF1 patients are usually affected by CNS neoplasms requiring contrast administration for a proper clinical evaluation, still limits the integration of ALS in clinical practice, indirectly strengthening the role of DSC-PWI as a noninvasive technique to evaluate cerebral perfusion in NF1 patients.

When we evaluated the possible correlation between cerebral perfusion data as measured by SPECT and MRI, we found that MRI-CBF was not useful to detect hypoperfused regions which were conversely identified by SPECT-CBF in 3 of 5 patients (60%). This discrepant result is in line with some [37] but not all [38] the previous studies that investigated the relationship between CBF measured by the two different imaging techniques. A possible explanation to this discrepancy could be researched in the different methodological principles underlying CBF measurements. In particular, while SPECT uses a lipophilic tracer to assess CBF which crosses the brain-blood barrier and permeate into the brain [39], gadolinium used in PWI-MRI is confined in cerebral vessels [40]. Given that the presence of collaterals introduces a well-known delay and dispersion of the contrast agent bolus in DSC-PWI, their presence introduce an underestimation of CBF (sometimes reported as reaching almost 40%), making this perfusion map less accurate [41].

In our cases, CBV was increased ipsilaterally to the occluded vessels in 5 patients (55%), in agreement with the previous reports [42–44]. Arterial dilation, secondary to cerebral hypoperfusion, represents the compensatory mechanism underlying the high signal observable in the cerebral subarachnoid spaces in MM patients. The discrepancy in subjects with preserved CBF, but increased CBV, may be explained by the presence of an early stage cerebral hemodynamic failure [45]. Indeed, when arterial stenosis reduces cerebral perfusion pressure, cerebral arterioles dilate to maintain CBF, leading to an increase in CBV with a preserved CBF. With further reduction in cerebral perfusion pressure, arterioles reach the maximum dilatation, and therefore, the CBV stop increasing and a decrease in CBF happens [45]. It has been suggested an inverse correlation between MTT and CVR as measured via SPECT with acetazolamide challenge [20], thus representing a noninvasive method to evaluate CVR. In this light, although being limited by a small number of samples, our results partly resemble those available in literature, with MTT maps correlating with SPECT data in all cases. As previously reported [46], these results suggest that MTT is sensitive to cerebral hemodynamic alterations, with its increase that have a significant reliability in the detection of CVR impairment.

Different limitations should be taken into account in this study, mainly related to the small sample size of our population. In particular, although some results are potentially interesting (namely, the correlation between MTT maps and SPECT data), we are aware that these findings are reported in a very small group of patients, that did not even allow for a proper statistical analysis. For this reason, future prospective studies, conducted on larger and heterogeneous populations, are strongly recommended, to further confirm our results.

Nevertheless, our results suggest that DSC-PWI could represent a useful noninvasive technique to evaluate hemodynamic impairment in MMS-NF1 patients. In particular, MTT maps have demonstrated a very good overlap with the CBF as measured using SPECT, thus encouraging for further studies to confirm a possible role of this perfusion parameter in the radiological evaluation of these patients.

## Electronic supplementary material

Supplementary Fig. 1 (PNG 1695 kb)

High resolution image (TIFF 2320 kb)

Supplementary Fig. 2 (PNG 1792 kb)

High resolution image (TIFF 1674 kb)

Supplementary Fig. 3 (PNG 859 kb)

High resolution image (TIFF 1151 kb)

Supplementary Fig. 4 (PNG 915 kb)

High resolution image (TIFF 1282 kb)

ESM 1 (DOCX 17 kb)

## Abbreviations

MRA: Magnetic resonance angiography
SPECT: Single-photon emission computed tomography
CBF: Cerebral blood flow
CBV: Cerebral blood volume
MTT: Mean transit time
TTP: Time to peak
ICA: Internal carotid artery
MCA: Middle cerebral artery
PCA: Posterior cerebral artery
MM: Moyamoya
EDAMS: Encephalo-duro-arterio-myo-synangiosis
