# Supplementary figures and images for: Multimodal evaluation of the cerebrovascular reserve in Neurofibromatosis type 1 patients with Moyamoya syndrome
Source: Neurol Sci. 2020 Jul 10;42(2):655–63. doi: 10.1007/s10072-020-04574-4 (PMC7843564; doi:10.1007/s10072-020-04574-4)

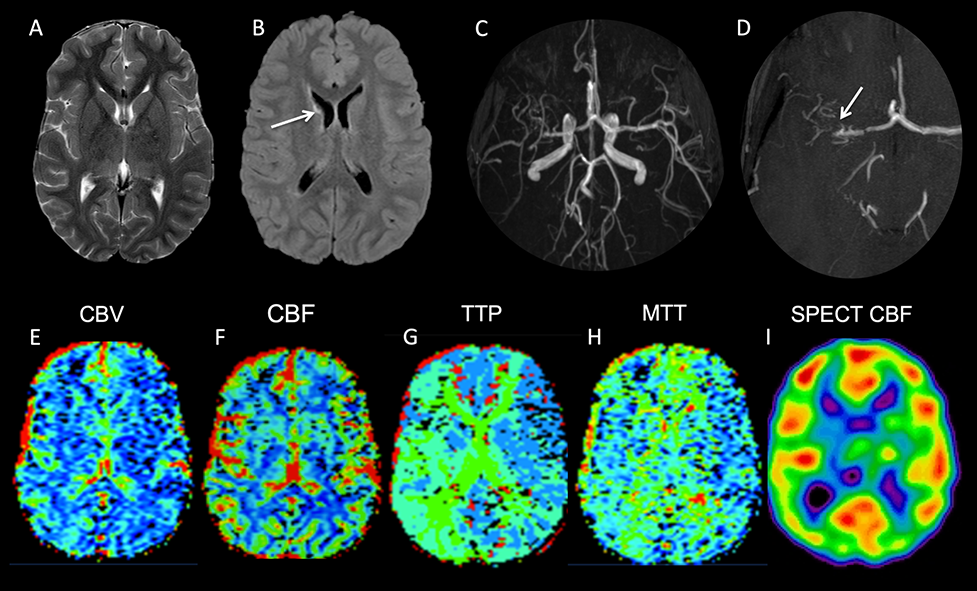

Supplement: Supplementary file 1 — (PNG 1695 kb) [file 10072_2020_4574_Fig4_ESM.png]

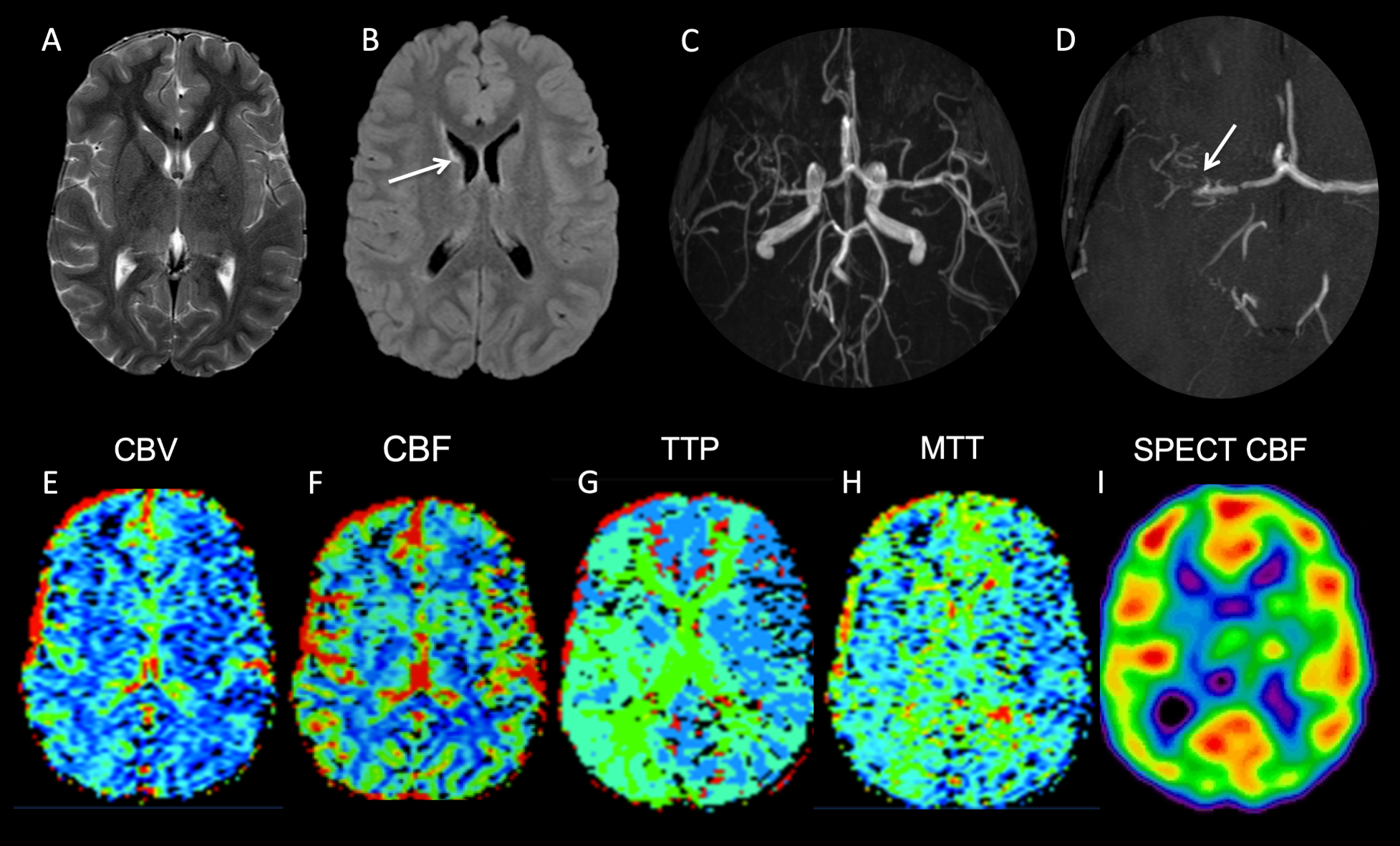

Supplement: Supplementary file 2 — High resolution image (TIFF 2320 kb) [file 10072_2020_4574_MOESM1_ESM.tiff]

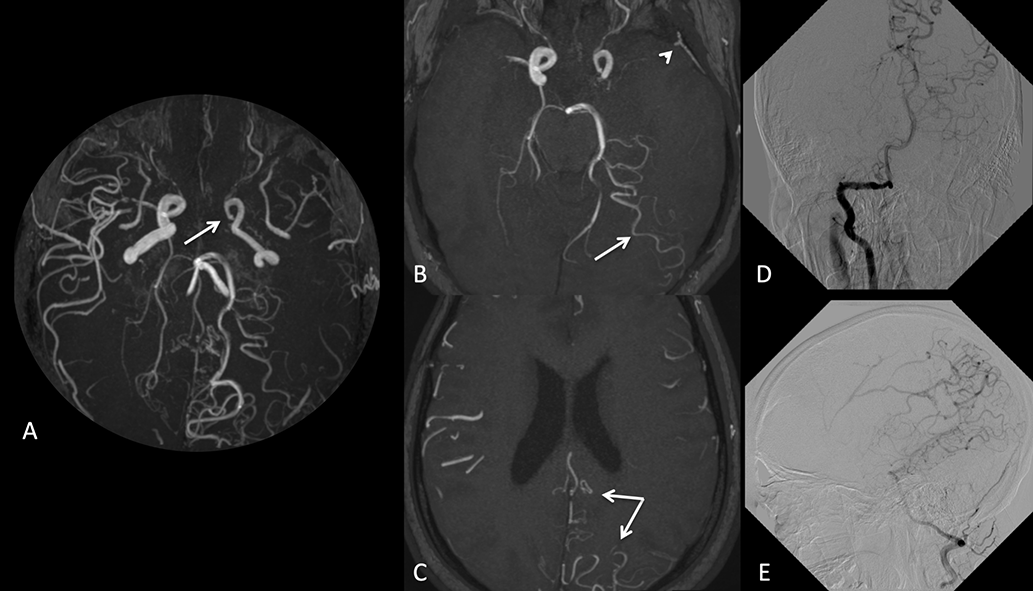

Supplement: Supplementary file 3 — (PNG 1792 kb) [file 10072_2020_4574_Fig5_ESM.png]

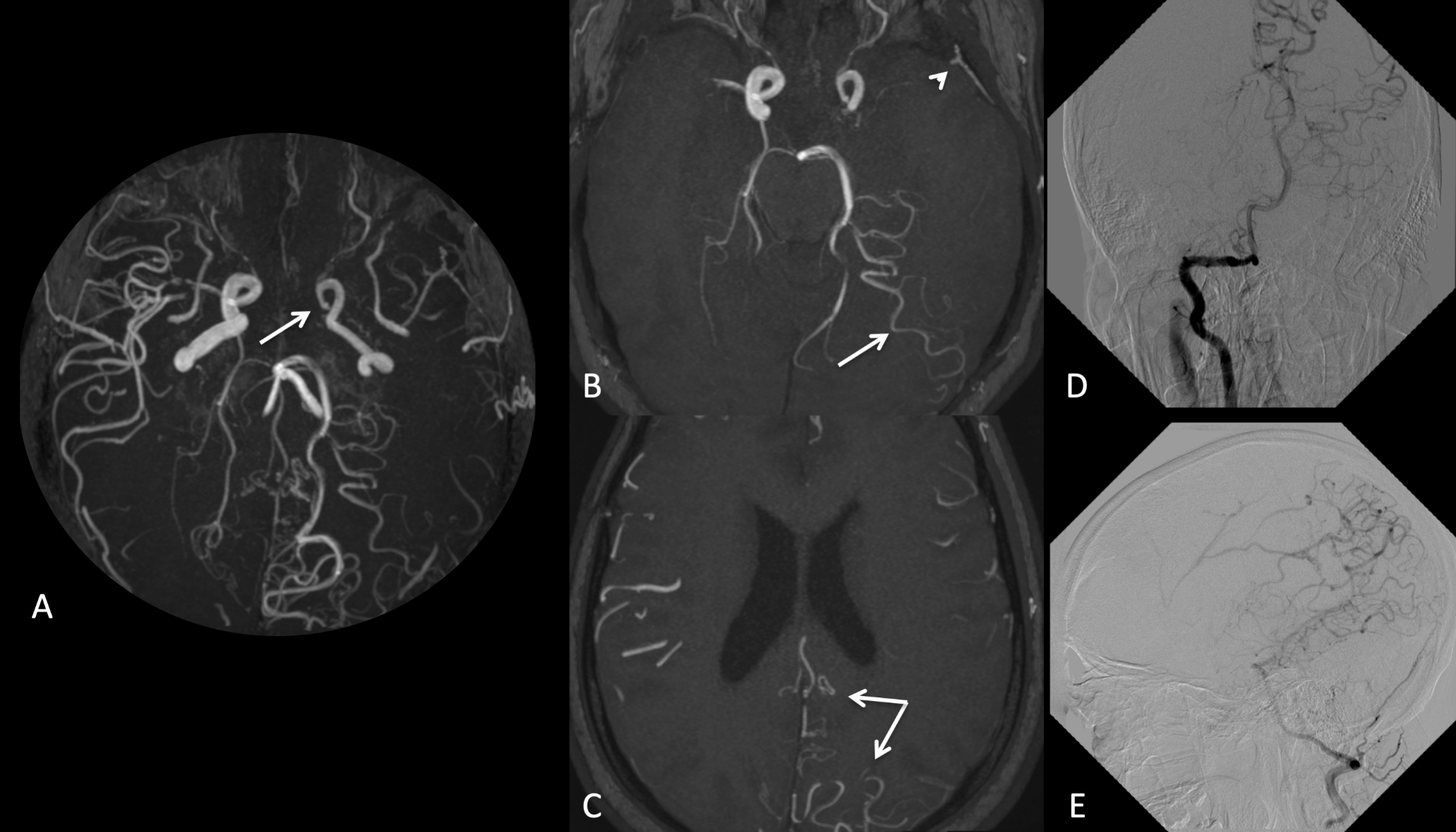

Supplement: Supplementary file 4 — High resolution image (TIFF 1674 kb) [file 10072_2020_4574_MOESM2_ESM.tiff]

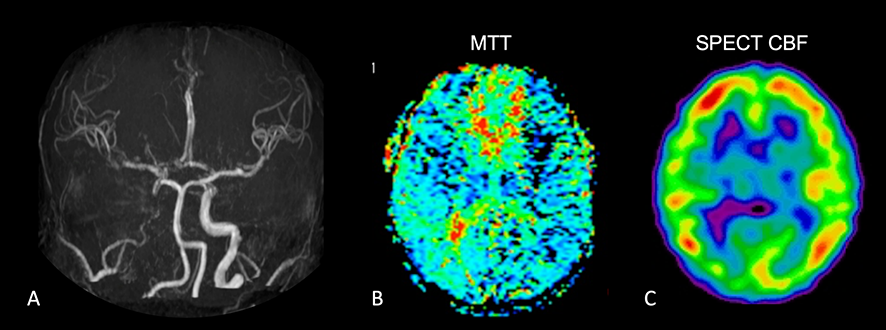

Supplement: Supplementary file 5 — (PNG 859 kb) [file 10072_2020_4574_Fig6_ESM.png]

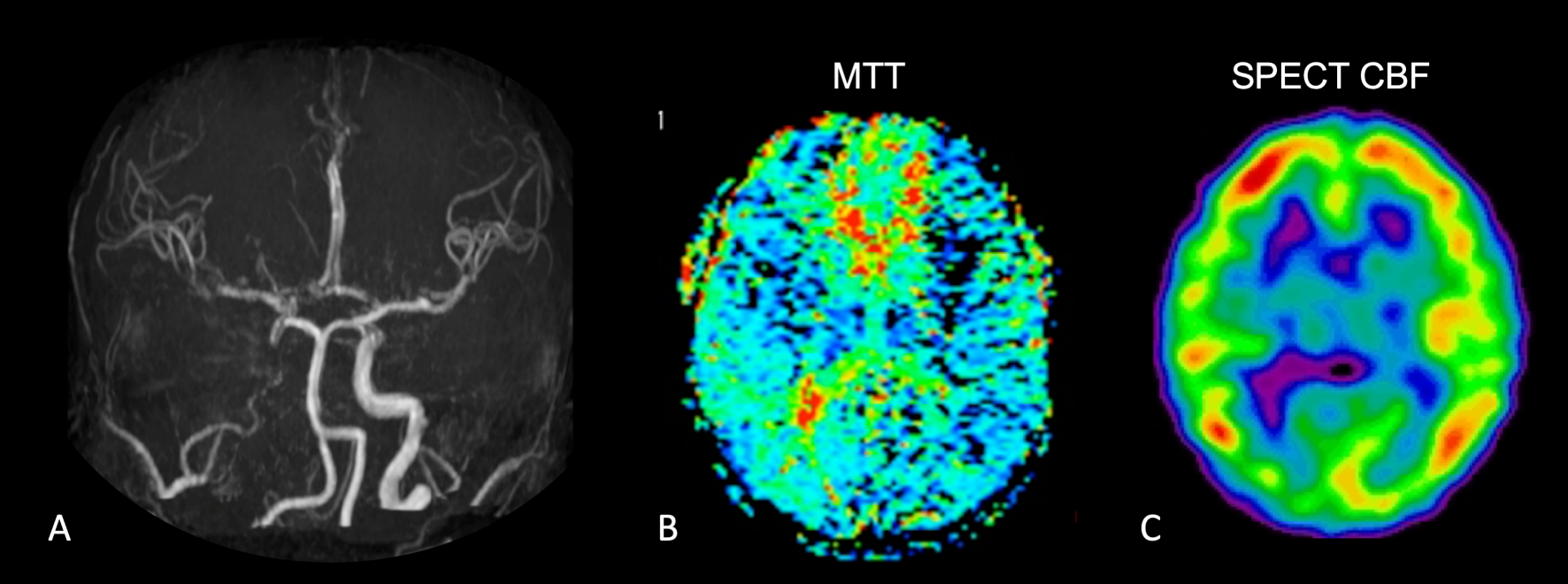

Supplement: Supplementary file 6 — High resolution image (TIFF 1151 kb) [file 10072_2020_4574_MOESM3_ESM.tiff]

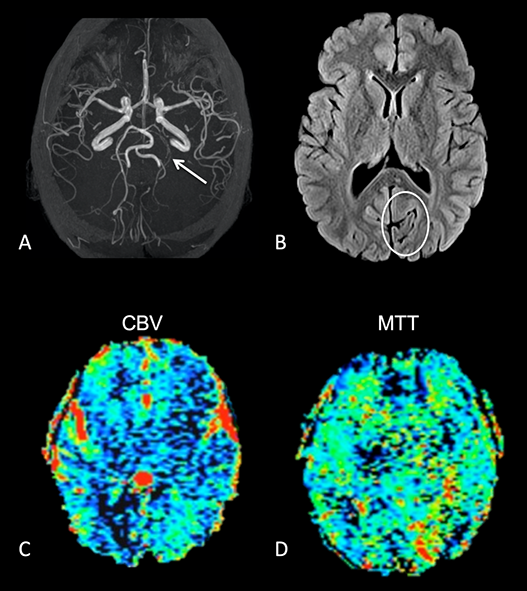

Supplement: Supplementary file 7 — (PNG 915 kb) [file 10072_2020_4574_Fig7_ESM.png]

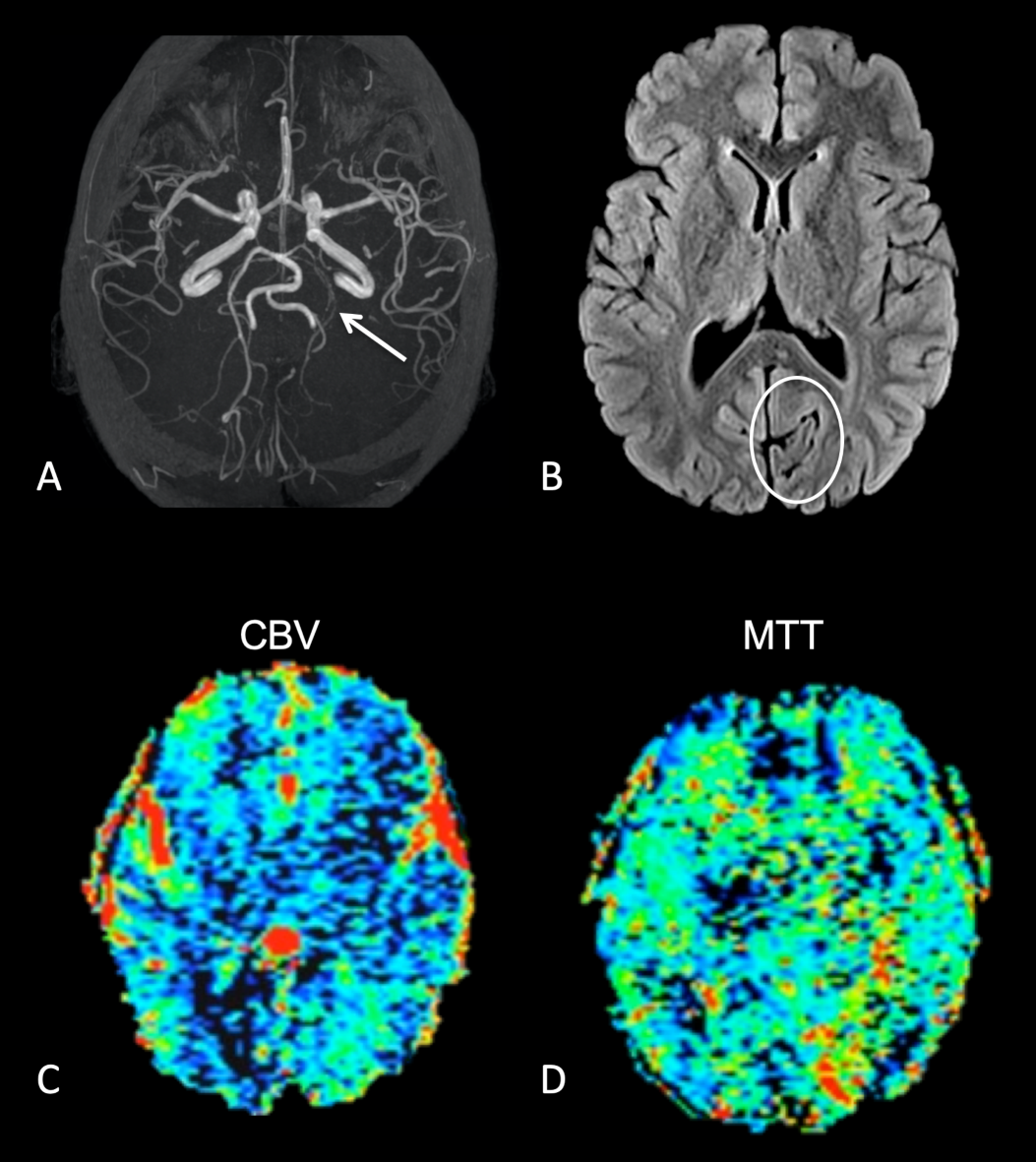

Supplement: Supplementary file 8 — High resolution image (TIFF 1282 kb) [file 10072_2020_4574_MOESM4_ESM.tiff]
