# Supplementary material for: Multimodal evaluation of the cerebrovascular reserve in Neurofibromatosis type 1 patients with Moyamoya syndrome
Source: Neurol Sci. 2020 Jul 10;42(2):655–63. doi: 10.1007/s10072-020-04574-4 (PMC7843564; doi:10.1007/s10072-020-04574-4)
Supplement: Supplementary file 9 — (DOCX 17 kb) [file 10072_2020_4574_MOESM5_ESM.docx]

**Multimodal evaluation of the cerebrovascular reserve in Neurofibromatosis Type 1 patients with Moyamoya Syndrome**

**CASE SERIES RESULTS**

*Patient #1*

A 11 years-old NF1 male presented with a voluminous plexiform neurofibroma of the left orbit and of the neck, migraine and dizziness.

Conventional MRI findings included the presence of "ivy sign" in the subarachnoid spaces of the left cerebral hemisphere on FLAIR images, as well as of the right frontal parasagittal region (Figure 2A-B). An extra- and intracranial left Internal Carotid Artery (ICA) occlusion was found, related to the presence of a voluminous ipsilateral trigeminal neurofibroma causing a complete infiltration of the ipsilateral cavernous sinus. Furthermore, several MM stenoses were noted at the level of the right distal ICA branches, both at A1, left P3 and P4 segments. Multiple thin collaterals were observed in the right sylvian fissure and in the quadrigeminal cistern (Figure 2C-D).

At the DSC-PWI evaluation, CBF was symmetrical, while CBV, MTT and TTP asymmetries were present (Figure 2E-H). In particular, CBV maps showed the presence of an increased blood volume in the left hemispherical subarachnoid spaces, while MTT map showed increased cortical values in the left parietal region, showing some overlap with the presence of the “ivy sign”. Finally, the TTP maps showed a significant increase affecting the entire left hemisphere.

At the SPECT images, rCBF findings confirmed the presence of the left cerebral hemisphere hypoperfusion showed at the DSC-PWI images (Figure 2I). Similarly, pre-surgical DSA confirmed MRA findings of several ICA stenoses.

The patient underwent EDAMS, after which migraine and dizziness gradually improved.

*Patient #2*

A 11 years-old female presented to our Institution with acute bilateral decreased visual acuity.

Conventional MRI data showed the presence of a chronic ischemic lesion of the right caudate nucleus, along with the presence of "ivy sign” in the ipsilateral temporo-sylvian and parietal sulci (Supplementary Figure 1A-B). On the same side, MRA demonstrated the presence of uniform caliber reduction of different ICA branches, with sub-occlusions of M1/M2 segments and MM collaterals in ipsilateral basal ganglia and in sylvian fissure (Supplementary Figure 1C-D).

At DSC-PWI, Patient 2 showed the presence of elevated CBV in the right posterior frontal, parietal and occipital regions, with preserved CBF values (Supplementary Figure 1E-F). MTT and TTP were clearly increased on the right hemisphere, including the ipsilateral caudate nucleus (Supplementary Figure 1G-H).

CBF measured at SPECT showed a similar pattern to those observed at MRI, with a mild hypoperfusion of the right temporo-occipital cortex (Supplementary Figure 1I). Presurgical DSA confirmed MRA findings, and right EDAMS was subsequently performed, with resolution of the visual symptoms. The patient is now asymptomatic.

*Patient #3*

This 11 years-old male presented with several voluminous neurofibromas of the posterior neck, and persistent cephalalgia.

On conventional MRI, no relevant findings were observed. On the left side, MRA showed stenosis of distal ICA branches, with occlusion of the Middle Cerebral Artery (MCA) and MM collaterals in the sylvian fissure, confirmed at the presurgical DSA (Supplementary Figure 2).

At DSC-PWI a subtle increase of the signal in the left lenticular nucleus, as well as in the ipsilateral frontal lobe, was noted on the CBV map. On the other hand, on TTP maps a delayed bolus arrival in the left cortical, subcortical and deep fronto-parietal and insular regions was present, while CBF and MTT maps showed no significant changes between the two hemispheres. In line with this last finding, a preserved rCBF was found at the SPECT maps.

Because of the persistent cephalalgia, left EDAMS was performed, with subsequent clinical remission.

*Patient #4*

A 14 years-old male presented with persistent migraine.

MRI showed the presence of a right optic nerve glioma, without further intracranial anomalies. MRA showed the presence of an occlusion of the right ICA, with the right MCA that was supplied by the anterior and posterior communicating arteries, in absence of MM collaterals (Supplementary Figure 3A).

DSC-PWI maps showed symmetric and preserved CBF and CBV, with increased MTT and TTP at the level of the right frontal and occipito-temporal areas (Supplementary Figure 3B), corresponding to an area of hypoperfusion at the rCBF obtained from SPECT data (Supplementary Figure 3C).

Despite the presence of clinical symptoms and the positive imaging findings, parents did not allow EDAMS. The patient is followed up with clinical examination every 6 months and annual MRI acquisition, with substantial clinical stability in the following 4 years.

*Patient #5*

This 16 years-old male presented to our Institution for intellectual disability.

Along with brain MRI, maxillo-facial district was also studied due to the presence of a right cheekbulky neurofibroma.

Occlusion of the right MCA with ipsilateral MM sylvian collateral arteries were evident at MRA, without further pathological findings on conventional MRI.

At DSC-PWI exam, a slight increase of TTP was found in the entire right cerebral hemisphere, in absence of any other DSC-PWI or SPECT abnormal finding.

Due to the substantial absence of clinical symptoms and imaging abnormalities, the patient was submitted to a strict clinical and MRI follow-up, being currently stable after 4 years.

*Patient #6*

This 12 years-old female presented with headache and left-sided paresthesia involving face, arm and leg.

Conventional MRI showed the presence of a pilocytic astrocytoma at the level of the left insular cortex. At MRA, the patient showed severe bilateral distal ICA stenoses (right > left), along with the occlusion of both MCAs and a very thin signal from ACAs.

On the left side, a significant number of MM vessels were found in the sylvian fissure, as well as in the posterior pericallosal region and at the level of the basal ganglia.

Interestingly, DSC-PWI did not show any cerebral flow asymmetries because of the bilateral involvement.

Given the severity of the clinical presentation, SPECT imaging was not acquired, and the patient was rapidly directed to surgery, where EDAMS was performed and after which neurological symptoms disappeared.

*Patient #7*

This 7 years-old female was followed with MRI in our Department due to three brain pilocytic astrocytomas previously diagnosed. At a follow-up MRI, without significant clinical changes, a small area of cortical and subcortical gliosis in the right occipital pole appeared, with mild enlargement of the right occipital horn and subarachnoid spaces. At MRA absent flow signal at the level of the right Posterior Cerebral Artery (PCA) and superior cerebellar artery (SCA) was detected, with sparing of the anterior circulation.

The evaluation of DSC-PWI maps showed decreased CBV and CBF values in the right cerebellar hemisphere and in the ipsilateral temporo-occipital white matter, coupled to an increase of MTT and TTP parameters. Nevertheless, in all DSC-PWI maps, thalamic perfusion was preserved and symmetric.

Due to the location and the substantial absence of clinical symptoms related to right posterior circulation stenosis, the patient was submitted to a strict clinical and MRI follow-up, being currently stable.

*Patient #8*

This subject wasa 11 years old male that underwent brain MRI because of clinical NF1 diagnosis, in absence of neurological symptoms.

On conventional MRI, mild occipital subarachnoid spaces enlargement was evident on FLAIR. At MRA, a significant left PCA stenosis was found, without ICA involvement (Supplementary Figure 4A-B).In line with this finding, the DSC-PWI maps showed at the level of the left occipital lobe an increased CBV, along with an increase of MTT and TTP parameters, without significant asymmetries of the CBF map (Supplementary Figure 4C-D).

The patient is followed up with clinical and MRI examination and is still asymptomatic.

*Patient #9*

This 21 years-old female had been long followed in our Department due to a severe psychomotor delay, epilepsy and left hemiparesis.

Previous MRI showed the presence of a chiasmatic glioma, treated with stereotacticradiotherapy 12years ago, along with a temporal cavernous malformation and a left trigeminal extracranial neurofibroma.

At MRA, an occlusion of the intracranial tract of the right ICA was present, along with a severe narrowing of the extracranial tract not undergoing irradiation (Figure 3A). Due to this carotid stenosis, the right frontal lobe was reduced in volume, also showing hyperintense signal on T2-weighted images (Figure 3B-C). Furthermore,asignificant FLAIR-weighted hyperintensity of the subarachnoid spaces (ivy sign)of the remaining right cerebral hemisphere was clearly recognizable, associated to ipsilateral leptomeningeal enhancement and MM vessels (Figure 3D).

At DSC-PWI examination, a significant reduction of both CBF and CBV maps at the level of the right frontal lobe was observed (Figure 3E-F), coupled to an increase of the MTT and TTP metrics (Figure 3G-H). Interestingly, the remaining right hemisphere also showed an increase of the MTT and TTP values, but coupled to a similar increase in CBF and CBV.

Unfortunately, in the following years a left cerebellar hemispheric glioblastoma was diagnosed, and the patient was lost at follow-up.

**FIGURES**

**Supplementary** **Figure 1**

Imaging findings in Patient #2.

A mild dilatation of the right hemispherical subarachnoid spaces secondary to hypoperfusion in evident on T2-weighted sequence (A), while FLAIR image shows a chronic ischemic lesion of the right caudate nucleus (B). MRA demonstrates uniform caliber reduction of the right ICA, together with sub-occlusion of M1/M2segments of MCA (C), with the presence of MM vessels in the ipsilateral sylvian fissure (D). On DSC-PWI, high CBV is evident in the right posterior frontal, parietal and occipital regions (E), while CBF results normal (F). TTP (G) and MTT (H) maps showed an increase affecting on the right side. SPECT (I) shows a mild hypoperfusion of the right temporo-occipital cortex.

**Supplementary** **Figure 2**

Vascular findings in Patient #3.

MRA (A-C) and DSA (D-E) show distal ICA stenosis on the left side with ipsilateral MCA occlusion. Middle meningeal artery (arrowhead on B) and PCA collaterals (arrows on B-C) hypertrophy are recognizable.

**Supplementary** **Figure 3**

Imaging findings in Patient #4.

MRA shows right ICA occlusion with the MCA supplied by the anterior and posterior communicating arteries (A).On the same side, DSC-PWI data showed an increase in MTT measures at the level of the right frontal and occipito-temporal areas (B), corresponding to an area of hypoperfusion on SPECT data (C).

**Supplementary** **Figure 4**

Imaging findings in Patient #8.

MRA shows left PCA stenosis (A) without carotid circulation involvement. Mild occipital subarachnoid spaces enlargement secondary to hypoperfusion in evident on FLAIR (B). An increased CBV (C) along with an increase of MTT (D) in the left occipital lobe were found at DSC-PWI.
